# The Toxicity Data Landscape for Environmental Chemicals

**DOI:** 10.1289/ehp.0800168

**Published:** 2008-12-22

**Authors:** Richard Judson, Ann Richard, David J. Dix, Keith Houck, Matthew Martin, Robert Kavlock, Vicki Dellarco, Tala Henry, Todd Holderman, Philip Sayre, Shirlee Tan, Thomas Carpenter, Edwin Smith

**Affiliations:** 1 National Center for Computational Toxicology, Office of Research and Development, U.S. Environmental Protection Agency, Research Triangle Park, North Carolina, USA;; 2 Office of Pesticide Programs, Office of Prevention, Pesticides, and Toxic Substances, U.S. Environmental Protection Agency, Arlington, Virginia, USA;; 3 Office of Pollution Prevention and Toxics and; 4 Office of Science Coordination and Policy, Office of Prevention, Pesticides, and Toxic Substances, U.S. Environmental Protection Agency, Washington, DC, USA;; 5 Office of Water, Office of Ground Water and Drinking Water, U.S. Environmental Protection Agency, Washington, DC, USA;; 6 Great Lakes National Program Office, U.S. Environmental Protection Agency, Chicago, Illinois, USA

**Keywords:** ACToR, carcinogenicity, database, developmental, hazard, HPV, MPV, pesticide, reproductive, toxicity

## Abstract

**Objective:**

Thousands of chemicals are in common use, but only a portion of them have undergone significant toxicologic evaluation, leading to the need to prioritize the remainder for targeted testing. To address this issue, the U.S. Environmental Protection Agency (EPA) and other organizations are developing chemical screening and prioritization programs. As part of these efforts, it is important to catalog, from widely dispersed sources, the toxicology information that is available. The main objective of this analysis is to define a list of environmental chemicals that are candidates for the U.S. EPA screening and prioritization process, and to catalog the available toxicology information.

**Data sources:**

We are developing ACToR (Aggregated Computational Toxicology Resource), which combines information for hundreds of thousands of chemicals from > 200 public sources, including the U.S. EPA, National Institutes of Health, Food and Drug Administration, corresponding agencies in Canada, Europe, and Japan, and academic sources.

**Data extraction:**

ACToR contains chemical structure information; physical–chemical properties; *in vitro* assay data; tabular *in vivo* data; summary toxicology calls (e.g., a statement that a chemical is considered to be a human carcinogen); and links to online toxicology summaries. Here, we use data from ACToR to assess the toxicity data landscape for environmental chemicals.

**Data synthesis:**

We show results for a set of 9,912 environmental chemicals being considered for analysis as part of the U.S. EPA ToxCast screening and prioritization program. These include high-and medium-production-volume chemicals, pesticide active and inert ingredients, and drinking water contaminants.

**Conclusions:**

Approximately two-thirds of these chemicals have at least limited toxicity summaries available. About one-quarter have been assessed in at least one highly curated toxicology evaluation database such as the U.S. EPA Toxicology Reference Database, U.S. EPA Integrated Risk Information System, and the National Toxicology Program.

The U.S. Environmental Protection Agency (EPA) has a significant interest in developing more efficient and informative toxicity determination approaches in part because of the large number of chemicals under its jurisdiction. Ultimately, it would be beneficial to characterize the toxicologic profiles of all chemicals in use in the United States. However, the size of this chemical universe [in excess of 75,000 chemicals, which is the estimated number in the Toxic Substances Control Act (TSCA 1976) inventory (U.S. EPA 2004b) makes this goal too difficult using current approaches to toxicity characterization that rely on extensive animal testing, cost millions of dollars, and can take 2–3 years per chemical. The International Life Sciences Institute/Health and Environmental Sciences Institute (ILSI/HESI) recently released several reports describing a more focused, tier-based approach for toxicity testing of agricultural chemicals, which would ultimately lead to the use of fewer animals (Barton et al. 2006; Carmichael et al. 2006). The National Research Council (NRC) recently released a report titled *Toxicity Testing in the 21st Century: A Vision and a Strategy* that outlines a much more ambitious and long-term vision for developing novel *in vitro* approaches to chemical toxicity characterization and prediction (NRC 2007) that would largely eliminate animal testing. The NRC report addresses several concerns about the current testing methods, specifically, the desire *a*) to reduce the number of animals used in testing, *b*) to reduce the overall cost and time required to characterize each chemical, and *c*) to increase the level of mechanistic understanding of chemical toxicity. The U.S. EPA and the National Institutes of Health (NIH) are actively pursuing approaches to implement ideas outlined in the NRC report (Collins et al. 2008).

Regardless of the level of quality of toxicology data on environmental chemicals, many chemicals lack significant amounts of data. In the United States and Canada, an estimated 30,000 chemicals are in wide commercial use, based on U.S. EPA and Environment Canada data (Muir and Howard 2006). The European Union’s Registration, Evaluation, and Authorization of Chemicals (REACH) program has recently released its first set of registered substances, which contains > 140,000 entries (REACH 2008). The exact number of chemicals in use is, in a sense, unknowable because it depends on where one sets the threshold of use and because use changes over time. The major point is that the number is relatively large and that only a relatively small subset of these chemicals have been sufficiently well characterized for their potential to cause human or ecologic toxicity to support regulatory action. This “data gap” is well documented (Allanou et al. 1999; Applegate and Baer 2006; Birnbaum et al. 2003; Guth et al. 2005; NRC 2007; U.S. EPA 1998).

The high cost and lengthy times associated with the use of animal testing to determine a chemical’s potential for toxicity make this strategy impractical for evaluating tens of thousands of chemicals, hence the large inventories of existing chemicals for which few or no test data are available. An alternative approach is to attempt to assess much larger numbers of chemicals by employing more efficient *in vitro* methods. One strategy applies a broad spectrum of relatively inexpensive and rapid high-throughput screening (HTS) assays to a large set of chemicals, followed by the use of these results to prioritize a much smaller subset of chemicals for more detailed analysis. The “prioritization score” for a chemical would be based on signatures, or patterns extracted from the HTS data, that are predictive of particular effects or modes of chemical toxicity. A comprehensive prioritization approach will also require the use of exposure and pharmacokinetic estimates, in addition to the intrinsic hazard information provided by *in vitro* assays. Chemicals of known toxicity make up the training and validation sets that are used to develop and validate these predictive signatures. HTS assays that yield data for the predictive signatures would then be run on chemicals of unknown toxicity (the test chemicals), and a prioritization score for those chemicals would be produced. The U.S. EPA has made a significant investment in this approach through the ToxCast research program (Dix et al. 2007). ToxCast is currently screening hundreds, and eventually thousands, of environmental chemicals using hundreds of HTS assays with the goal to develop predictive toxicity signatures, and is using these signatures to prioritize chemicals for further testing. In this context, the term “environmental chemicals” refers primarily to pesticides and industrial chemicals that are used or produced in large enough quantities to pose potential for human or ecologic exposure [largely the high-production-volume (HPV) and medium-production-volume (MPV) chemicals described below]. However, a number of environmental chemicals that are captured in our analysis are food ingredients or naturally occurring human metabolites. We included many of the former because they are classified as inert ingredients in pesticide products.

In this article we address two key aspects of this chemical screening and prioritization process. The first is the definition of a set of chemicals of interest to a screening program, based on their widespread use or other potential for significant human exposure, or the current availability of toxicity information that can be used in building screening models. Some widely used but as yet uncharacterized chemicals may not be good candidates for screening because their physical–chemical properties make them impractical to test in *in vitro* assays (e.g., insoluble or highly volatile compounds), whereas other substances that we define as environmental chemicals are regarded to be safe under intended use situations and may not require further testing, but can serve as negative controls. For instance, a subset of pesticide inert ingredients are also on the U.S. Food and Drug Administration (FDA) Generally Recognized as Safe chemical list. As a further example, some “chemicals” that are listed as pesticide inert ingredients are common foods, such as milk.

The second objective is the characterization of the sources and amount of reliable *in vivo* toxicology data that can be used for developing and validating screening models in programs such as ToxCast. A significant amount of high-quality toxicity data are needed to train and validate *in vitro*–based models for predicting chemical hazard. Equally important is the presence of both negative and positive examples for each toxicity end point to be modeled. In addition to the sets of environmental chemicals described here, pharmaceutical compounds are another source of detailed animal and human toxicology data.

The sets of chemicals on which we have focused are the HPV and MPV chemicals from the TSCA inventory, pesticide and antimicrobial active and inert ingredients, known drinking water contaminants, hazardous air pollutants (HAPs) and certain defined classes of chemicals of interest, including the U.S. EPA’s Toxics Release Inventory (TRI), Integrated Risk Information System (IRIS), and the first set of chemicals to be tested through the Endocrine Disruptor Screening Program (EDSP). The TRI, drinking water contaminant, and EDSP chemicals are largely included in the TSCA inventory and pesticide active and inert ingredient lists. By combining these sources, we define a set of 9,912 chemicals. Below we describe in detail the process we used to arrive at this number. At present, we have limited the scope of *in vivo* toxicology data to that which is relevant to human health, as opposed to ecotoxicity. An equivalent analysis for the ecotoxicity data landscape will be carried out in the future.

To support a data-intensive analysis of environmental chemicals, we have developed a system called ACToR (Aggregated Computational Toxicology Resource) (Judson et al. 2008; U.S. EPA 2008a), which is a database holding essentially all publicly available information on chemical identity, structure, physical–chemical properties, *in vitro* assay results, and *in vivo* toxicology data. All of the data described in this article have been collected in ACToR.

## Target Chemicals for Analysis

The U.S. EPA has authority to review and/or regulate a large number of chemicals under a variety of statutes, including those governing the manufacture, import, sale, and use of pesticides and industrial chemicals. The large numbers of chemicals on various U.S. chemical inventories, and the limited toxicity information for many of these, have already been stated as the driver for the need to set priorities for additional testing. Because this universe of chemicals is so large, it is even necessary to prioritize what goes into a science-based prioritization approach such as ToxCast. In this article we focus on chemicals that are of interest because *a*) they are known to be bioactive (e.g., pesticide active ingredients), *b*) they are manufactured or used in large quantities (HPV and MPV chemicals), or *c*) many people may be exposed to them on a routine basis (e.g., drinking water contaminants). We include both largely uncharacterized chemicals and chemicals for which significant toxicology information is already available (e.g., pesticide active ingredients, IRIS chemicals, and chemicals on the TRI). The well-characterized chemical groups are important because these allow us to develop and validate predictive models for prioritization of the remaining, largely uncharacterized chemicals.

Based on these criteria, we focused on sets of chemicals that are defined in the remainder of this section. Some of these lists are not static, so we have chosen versions available as of a specific date. For each of the lists, we describe the rules for inclusion and provide the total number of chemicals used for the current evaluation. “Official” versions of these lists are updated and posted to the relevant U.S. EPA websites only every 2 or more years, so in several cases, we have extracted more current snapshots of the lists from internal U.S. EPA databases. Many of the chemicals we included in this analysis are complex mixtures. Additionally, these lists have significant overlap; for instance, some pesticide active ingredients are also HPV chemicals. Finally, to be included in the current ACToR inventory, a chemical must be identified by a Chemical Abstracts Service Registry Number (CASRN).

Possible later extensions of this analysis could consider chemicals with lower production volumes or lower exposure potential than those considered presently. These would include the Canadian Domestic Substances List (DSL), which includes approximately 30,000 chemicals, and the large collection of chemicals to be analyzed under the REACH program. REACH is still in the process of defining its target list, but an estimated 30,000 chemicals will be included. Many of the Canadian DSL and REACH chemicals have U.S. use and/or production levels below the cutoffs used for the present analysis. Note, however, that the Canadian DSL and the chemicals we considered here significantly overlap. Additionally, pharmaceutical compounds will be included in the future because of the corresponding wealth of both animal and human toxicology data.

### The TSCA Inventory and Inventory Update Reporting (IUR)

In 1977, the U.S. EPA published a rule to assemble an inventory of chemical substances currently in commerce. This inventory, commonly referred to as the TSCA Inventory, is the basis for the U.S. EPA’s Existing Chemicals Program. Starting in 1986, the Inventory was periodically updated using the IUR regulation. The TSCA Inventory is composed of approximately 85,000 chemical substances (U.S. EPA 2004b), including both substances that are nonconfidential and those claimed to be confidential business information (CBI) under TSCA. Originally, the IUR was updated on a 4-year cycle, but starting with the 2006 IUR, it will be updated on a 5-year cycle. The IUR reporting requirements depend on the volume of the chemical that is produced as well as certain exemptions. Hence, the IUR list is a subset of the larger TSCA inventory. Before 2006, the IUR contained organic chemicals manufactured or distributed in the United States in amounts ≥ 10,000 lb/year. The 2006 IUR regulation requires manufacturers and importers of certain chemical substances to report site and manufacturing information for chemicals manufactured or imported in amounts of ≥ 25,000 lb at a single site. Additional information on domestic processing and use must be reported for chemicals manufactured in amounts of ≥ 300,000 lb at a single site. The full inventory, including both confidential and nonconfidential substances, is maintained by U.S. EPA and Chemical Abstract Service and is not available to the public. The nonconfidential or “public” inventory is published periodically, usually after each IUR cycle. We have included the 2002 version of the public TSCA inventory in our analyses. This list is available from the U.S. EPA Substance Registry System (U.S. EPA 2008q). This list contains 65,513 chemicals indexed by CASRN. Note that this number differs from the 75,000 quoted elsewhere because this is the publicly released list and excludes chemicals added under the claim of CBI.

### HPV chemicals

The U.S. HPV chemicals are those manufactured in or imported into the United States in amounts ≥ 1 million lb/year. The U.S. EPA HPV list is fluid, changing to some degree with each IUR cycle. Our current list contains 2,539 chemicals (U.S. EPA 1990). We also include two important subsets of the HPV list.

### U.S. EPA HPV Challenge

The HPV Challenge Program chemical list consists of all the HPV chemicals reported during the 1990 IUR reporting year. Inorganic chemicals and polymers, except in special circumstances, were not included in the HPV Challenge Program. Our version of the HPV Challenge list contains 1,973 chemicals (U.S. EPA 1990).

### U.S. EPA HPV information system

These are chemicals with data submitted under the HPV Challenge Program for which “Robust Summary” data have been entered into the U.S. EPA HPV information system (HPVIS; U.S. EPA 2008l). There are 991 chemicals from HPVIS with information in ACToR.

### MPV chemicals

Another set of industrial chemicals of interest are the non-HPV chemicals included in the TSCA IUR list. These are the chemicals exceeding a reporting threshold of 10,000 lb/year before 2006, and 25,000 lb in 2006 and beyond, but < 1 million lb/year. The 2002 IUR list contains 5,375 MPV chemicals (U.S. EPA 2004a, 2004b). The updated, draft 2006 IUR list contains approximately 3,668 MPV chemicals that are not CBI; the 2006 IUR public list will be released by the U.S. EPA in 2009.

### Pesticides and antimicrobials

This category covers a wide range of substances. Chemicals regulated as part of the U.S. EPA pesticide program are generally classified as “active” or “inert.” The active ingredients are further classified by whether they are targeted at microbes (antimicrobials) or complex organisms (pesticides). Additionally, all pesticide compounds (conventional actives, antimicrobials, and inert ingredients) are classified by whether or not they have food-use tolerances or tolerance exemptions. Finally, one can classify these chemicals by whether or not they are in use in significant quantities. Here we rely on the Office of Pesticide Products Information (OPPIN) system of the U.S. EPA to extract lists of chemicals. OPPIN is not publically accessible. From this, we have drawn the following subsets:

- Conventional Pesticide Actives: (EPA OPPIN pesticide active): active pesticide ingredients (834 chemicals)
- Antimicrobial Actives (EPA OPPIN antimicrobial active): active ingredients used against microbes (337 chemicals)
- Pesticide inert ingredients: an inert ingredient means any substance, other than an active ingredient, that is intentionally included in a pesticide product. Inert ingredients have a number of uses, for instance, as a solvent, as an aid in increasing the pesticide product’s shelf life, or as an agent to protect the pesticide from degradation due to exposure to sunlight. We used two sources: *a*) U.S. EPA OPPIN inert ingredients (the complete OPPIN list containing 3,532 chemicals); and *b*) U.S. EPA inert nonfood ingredients [a list of inert pesticide ingredients classified by hazard potential, not approved for food contact use, available from the U.S. EPA’s Office of Pesticide Programs (OPP) website (3,492 chemicals) (U.S. EPA 2008n)]
- Pesticide ingredients with food-use tolerances or tolerance exemptions (U.S. EPA OPPIN food use) (1,320 chemicals)

### U.S. EPA TRI

The Emergency Planning and Community Right-to-Know Act of 1986 (EPCRA 1986) requires businesses to report the locations and quantities of chemicals stored on-site to state and local governments in order to help communities prepare to respond to chemical spills and similar emergencies. EPCRA requires U.S. EPA and the states to annually collect data on releases and transfers of certain toxic chemicals from industrial facilities, and to make the data available to the public in the TRI. In 1990 Congress passed the Pollution Prevention Act of 1990 (Pollution Prevention Act 1990), which requires that additional data on waste management and source reduction activities be reported under the TRI. The U.S. EPA compiles the TRI data each year and makes these data available through several data access tools, including their website (U.S. EPA 2008p). Our analysis includes 636 chemicals from TRI.

### Drinking water contaminants

The U.S. EPA develops drinking water standards and identifies lists of potential drinking water contaminants because they are anticipated to occur in drinking water supplies and may have adverse health effects. The lists tracked in the present analysis are the U.S. EPA’s Drinking Water Standards and Health Advisory Chemicals (DWSHA; 200 chemicals) and the Candidate Chemical Lists [CCLs: U.S. EPA CCL1, U.S. EPA CCL2, and U.S. EPA draft CCL3, which include 47, 39, and 92 chemicals, respectively (U.S. EPA 2008e)]. We also included the Preliminary CCL (PCCL) listing of the 528 chemicals that the U.S. EPA evaluated during the development of draft CCL3 (U.S. EPA 2008d). The U.S. EPA PCCL was derived from a collection of approximately 6,000 chemicals analyzed by the U.S. EPA’s Office of Water, and the PCCL was selected from these 6,000 chemicals based on available health effects and occurrence data (U.S. EPA 2008c).

### U.S. EPA Great Lakes National Program Office

A set of 429 candidate persistent, bioaccumulative toxicants (PBTs) compiled by the U.S. EPA Great Lakes National Program Office (GLNPO) are included in the present analysis (Muir and Howard 2006). These are designated as U.S. EPA GLNPO PBT chemicals.

### U.S. EPA HAPs

This is a list of chemicals that are under review by the U.S. EPA specified in the Clean Air Act Amendments of 1990. These chemicals include volatile organic chemicals, chemicals used as pesticides and herbicides, inorganic chemicals, and radionuclides. Many of these chemicals are used for a variety of purposes in the United States today. Other chemicals, although not in use today, were used extensively in the past and may still be found in the environment. We include a total of 185 chemicals from this source.

### EDSP chemicals

A variety of chemicals have been found to disrupt the endocrine systems of animals in laboratory studies, and compelling evidence shows that endocrine systems of certain fish and wildlife have been affected by chemical contaminants, resulting in developmental and reproductive problems. Based on this and other evidence, Congress passed the Food Quality Protection Act of 1996, which requires that the U.S. EPA test for the potential estrogenic effects in humans. Subsequently, a U.S. EPA advisory committee recommended that this be expanded to include effects occurring via androgen and thyroid mechanisms and potential for effects on ecologic species. We have included the 73 chemicals that were listed to be screened under Tier 1 of the U.S. EPA EDSP (U.S. EPA 2007a).

### ToxCast phase I chemicals

ToxCast is a U.S. EPA program designed to apply HTS, high-content screening and genomics techniques to the screening and prioritization of environmental chemicals (Dix et al. 2007). Phase I of this program is screening 309 unique chemicals, most of which are pesticide active ingredients. (One of the ToxCast chemicals has no CASRN, so we do not include it in the analyses below.) This chemical listing is available for download from the ToxCast or U.S. EPA Distributed Structure-Searchable Toxicity Data Network (DSSTox) websites (U.S. EPA 2008k, 2008o).

### Toxicology Reference Database

This is a collection of summary *in vivo* toxicology data, currently focused on pesticide active ingredients. Data on pesticide actives is collected and summarized from U.S. EPA OPP data evaluation records (DERs), which are summaries of guideline studies required before approval of new pesticide active ingredients. The Toxicology Reference Database (ToxRefDB) provides the toxicology data required to link *in vitro* assays from ToxCast with *in vivo* toxicity end points (Martin et al. 2008). ToxRefDB will eventually contain information on most of the pesticide active chemicals of ToxCast phase I and will later expand to include toxicity data on additional pesticide and nonpesticide chemicals. The current database contains information on 431 chemicals. In addition to data derived from pesticide DERs, ToxRefDB will contain data from other primary *in vivo* toxicology sources.

### U.S. EPA Integrated Risk Information System

The collection of chemicals subject to evaluation by the U.S. EPA Integrated Risk Information System (IRIS) program make up three major lists: the main U.S. EPA IRIS set (U.S. EPA 2008f), for which evaluations are currently available (535 chemicals); the U.S. EPA IRIS nominations (U.S. EPA 2008g; currently 20 chemicals nominated for inclusion); and the U.S. EPA IRIS queue (U.S. EPA 2008h), which are chemicals in queue to have IRIS reports written (68 chemicals).

### Target collection summary

The total number of chemicals (defined by unique CASRN) in this set of collections comes to 9,912. Table 1 shows the overlap matrix between these target chemical lists. The sum of the number of chemicals in the individual lists is 23,985. This number drops to 9,912 once we remove overlaps. For instance, 720 chemicals are on the U.S. EPA HPV and on the U.S. EPA OPPIN inert ingredients lists. From the U.S. EPA CCL3 list, 29 of 92 are also HPV chemicals. Interestingly, in a few cases no overlap occurs between pairs of lists. Two instances are the lack of overlap between the U.S. EPA CCL1 and CCL2 lists and the U.S. EPA GLNPO PBT list.

## Information Sources

The information that is available on the target chemicals can be divided into several assay categories. The sources for each of these types of data are available online at http://www.epa.gov/ncct/toxcast/.

### Chemical structures

We have compiled structures for most of the defined compounds (as opposed to mixtures) in the target lists. For subsets of chemicals, structures have been hand curated and quality reviewed as part of the U.S. EPA DSSTox program (Richard et al. 2008). We took the remaining structures from a variety of sources, including PubChem [National Center for Biotechnology Information (NCBI) 2008], the National Cancer Institute’s Chemical Structure Lookup Service (National Cancer Institute 2008), and the U.S. EPA Substance Registry System inventory. In many cases, structures were derived from Simplified Molecular Input Line Entry Specification (SMILES) codes (Daylight Chemical Information Systems, Inc. 2008). At present, we have chemical structures for 7,099 of the 9,912 target chemicals. We lack structure information for many chemicals because many substances on these lists are mixtures, sometimes relatively simple ones for which representative structures could be designated (e.g., “sulfuric acid, mono-C_14_–_18_^−^ alkyl esters, sodium salts”), and sometimes very complex mixtures (agar, sesame oil).

### Physical–chemical properties

We used U.S. EPA’s EPISuite (U.S. EPA 2007c) set of programs to calculate physical–chemical properties for a subset of chemicals. The input to EPISuite is a list of SMILES codes. Several EPISuite programs were used including KOWWIN [estimates the logarithmic octanol–water partition coefficient (logP, also sometimes called log *K*_ow_) of organic compounds (Meylan and Howard 1995)], MPBPWIN [estimates the boiling point (at 760 mm Hg), melting point, and vapor pressure of organic compounds (Stein and Brown 1994)], WATERNT (estimates the water solubility of organic compounds at 25°C; Meylan and Howard 1995), and WSKOWWIN (estimates the water solubility of an organic compound using the compounds log octanol–water partition coefficient; Meylan et al. 1996). The properties we use are molecular weight (MW), logP, boiling point, melting point, vapor pressure, phase at 25°C, and molar water solubility. EPISuite reports (and we) use experimental values when available.

### Biochemical (in vitro or cell-based) assay data

For a subset of the chemicals of interest, *in vitro* (biochemical) or cell-based assay data are currently available. This can include receptor binding, enzyme inhibition, or cytotoxicity. The major sources of these data are PubChem and the National Institute of Mental Health’s Psychoactive Drug Screening Program *K_i_* Database (Roth and Lopez 2008).

### In vivo toxicology assay data (tabular)

We derived these data from guideline (or equivalent) toxicology studies from which the primary or secondary data are available. For our purposes, the main sources of this primary data are the National Toxicology Program (NTP), U.S. EPA OPP (through ToxRefDB; Martin et al. 2008), the U.S. EPA’s HPVIS, and the FDA. The FDA data we used here came from the following databases: *a*) FDA Generally Recognized as Safe list; *b*) FDA Cumulative Estimated Daily Intake/Acceptable Daily Intake Database; *c*) FDA Everything Added to Food in the United States database; and the *d*) FDA List of “Indirect” Additives Used in Food Contact Substances. We compiled our tabular primary data largely through the ToxRefDB database (Martin et al. 2008) and the DSSTox programs (Richard et al. 2006). HPVIS is a special case because it includes both primary and secondary data, often provided in summary by sponsors, with data derived either from the open literature or from sponsor-derived study reports. The database captures so-called “Robust Summaries.” Examples of secondary tabular *in vivo* toxicity data are the Carcinogenic Potency Database (Gold et al. 2001), U.S. EPA IRIS reports, National Library of Medicine (NLM) TOXNET databases (Hazardous Substances Data Bank and Chemical Carcinogenesis Research Information System), and California EPA. Data from a number of these secondary sources have been tabulated and made available through the DSSTox program (Richard et al. 2007). Types of tabular information that are captured in the DSSTox program include high-level summary results such as food-use tolerances, LOAELs and NOAELs (lowest and no observed adverse effect levels), and reference doses, as well as highly detailed data such as the per-animal or group-level results of toxicology studies. Cell-based genotoxicity is currently captured under this category because it co-occurs with rodent carcinogenicity data in current ACToR data sources.

### In vivo toxicology text reports via URL

Much of the publicly available *in vivo* toxicology data are in the form of narrative reports from which detailed tabular data may or may not have been extracted. Examples are the original NTP, IRIS, and Screening Information Data Sets (SIDS) reports, the latter from the Organization for Economic Cooperation and Development (OECD) HPV Programme. We also included the International Agency for Research on Cancer (IARC) and Agency for Toxic Substances and Disease Registry (ATSDR) study reports in this set. These reports contain quantitative and categorical data, but for most of these sources, the data provided are not easily extractable. All of the studies we used here are accessible via the Web. Information can be extracted from these reports on a case-by-case basis.

### In vivo toxicology summary calls

Several sources have made definitive calls concerning particular modes of toxicity, for instance, labeling chemicals as being human carcinogens or developmental toxicants. These calls are made by experts using data from the detailed toxicity reports described previously. Although the calls are subject to debate by experts, they provide a useful source of data for training prioritization models. This information is typically categorical. Examples of summary calls are cancer potential determinations of the California EPA (2008), the NTP Report on Carcinogens (NTP 2008b), NTP Center for the Evaluation of Risks to Human Reproduction (NTP 2008a), and the U.S. EPA OPP cancer classifications (U.S. EPA 2007b).

### Regulatory listings

By law, the U.S. EPA and some state agencies maintain a number of lists of chemicals that are of toxicologic concern. The presence of a chemical on one of these lists indicates that toxicity data are available. For the present analysis, we derived these lists from the U.S. EPA Substance Registry System (U.S. EPA 2008j).

### Phenotypes

Above we have described the information types of the data rather than the disease or toxicology categories. Where possible, assays or data sources have also been labeled by appropriate disease or toxicology categories, and we label these categories as “phenotypes.” The set of phenotypes implemented in ACToR span traditional toxicology study areas. The subset of phenotypes we use here are general hazard, carcinogenicity, genotoxicity, developmental toxicity, reproductive toxicity, and chronic toxicity. Other toxicity phenotypes are represented in ACToR, but for small numbers of chemicals. Many data sources, especially the toxicology summary reports, contain information on multiple types of toxicity or end points. In this category, we have included only IRIS, NTP, ToxRefDB, and U.S. EPA and OECD HPV SIDS reports because they can be assumed to have covered a defined standard set of areas of toxicity for most chemicals. “Hazard” is a very broad phenotype category that can include assays derived from acute and sub-chronic rodent studies at one end or material safety data sheets at the other. We further track information on food safety assessments, as provided by the FDA (FDA 2006, 2007, 2008). In addition, the U.S. EPA sets food-use tolerances (or tolerance exemptions) for a subset of pesticide ingredients. There is a significant overlap between chemicals regulated by the U.S. EPA and those analyzed by the FDA. It is obviously of great value to have both positive and negative toxicity information for all of the phenotypes, and both types were captured where they were available.

Several reviews of the toxicology data landscape have described sources of data that are included in ACToR. Yang et al. (2006a, 2006b) have recently published two such reviews. In 2001 and 2002, several review papers were published surveying the landscape of toxicity data available on the Internet (Brinkhuis 2001; Felsot 2002; Junghans et al. 2002; Patterson et al. 2002; Polifka and Faustman 2002; Poore et al. 2001; Richard and Williams 2003; Russom 2002; Winter 2002; Wolfgang and Johnson 2002; Young 2002).

We provide a summary of the sources of toxicology data we used in this analysis, available online at http://www.epa.gov/ncct/toxcast/. In the simplest case, each toxicology source is a single assay in the ACToR database. (There are multiple exceptions; e.g., DSSTox and NTP each contribute multiple assays.) For each assay, we list the short name, a description, the institutional source, the number of chemicals covered, the types of information provided, and a URL. There were 22 sources from which target screening chemicals were taken, 47 sources of toxicology data, and 48 lists of chemicals covered by regulations.

## Data Collection and Integration: ACToR

All of the data for this analysis are collected in the ACToR system (Judson et al. 2008; U.S. EPA 2008a). The organizing principles for the design of the chemical/assay system are largely derived from the PubChem project, which captures chemical structure and HTS information on millions of chemicals in its role as the main data repository for the NIH Molecular Libraries Roadmap (Austin et al. 2004). PubChem characterizes data in terms of “substances” (the actual chemical on which one performs an experiment as defined by the data source), “compounds” (the idealized structures of chemicals), and assays (data generated on substances). ACToR collects these same three main types of data: substances, indexed by substance identifier (called the SID); compounds (i.e., chemical structures) indexed by compound identifier (called the CID); and assays, indexed by assay identifier (called the AID). A substance is a single chemical entity from one data source and often corresponds to the physical substance on which some experiment was performed. A compound is a chemical entity that corresponds to a unique chemical structure. Because a substance is defined as being specific to both data source and experiment, many substances (SIDs) may map to a single compound (CID). An assay, indexed by AID, represents a specific type of test data associated with one or more substances. In ACToR, a substance is minimally characterized by a data-collection–specific SID and a chemical name. Most often, the substance will also have synonyms, a CASRN, and several other parameters. A compound always has an associated chemical structure and a data-collection–specific CID, in addition to optional parameters derived directly from chemical structures, such as SMILES (Daylight Chemical Information Systems, Inc. 2008) and International Chemical Identifier [International Union of Pure and Applied Chemistry (IUPAC) 2008)] linear chemical structure representations and MW. Note that because ACToR is in essence a “super-aggregator,” pulling in large external data collections, it also stores the source-labeled SIDs and CIDs from each independent collection (e.g., PubChem CID, DSSTox CID).

In ACToR, as in DSSTox, data on chemicals across data collections are aggregated using the concept of a generic chemical. Because most environmental chemicals, along with their related toxicity data, are indexed by CASRN, which can be thought of as a source-independent test SID, ACToR aggregates information based on this identifier. A generic chemical is defined by a CASRN, a preferred name (typically a common name rather than an IUPAC or other systematic name), and an optional ACToR CID. Some sources (in particular, the FDA and NTP) have provided CASRN-like identifiers for some compounds, and these are used in ACToR in place of the CASRN. All data on all substances sharing a particular CASRN are attached to the corresponding generic chemical. In particular, a generic chemical will inherit all names attached to substances with the corresponding CASRN as synonyms.

In ACToR, an assay is a generic collection of data values associated with a set of substances and (potentially) compounds (i.e., chemical structures). An assay has a unique AID, a name, an assay category, and, optionally, one or more “phenotypes.” Table 2 lists the assay categories (major types of assays). Assay phenotypes are linked to high-level classes of toxicity testing such as carcinogenicity or reproductive or developmental toxicology. This allows quick searching of the database to find all assays that pertain to that high-level toxicology concept. The concept of an assay as implemented in ACToR is purposely broad so as to capture any information potentially relevant to understanding toxicity and evaluating risk for environmental chemicals. An assay can also have one or more components, which are separate data fields that naturally fall together into an assay (e.g., the binding constant to a receptor at different concentrations). Each component is defined by an assay component identifier, the corresponding AID, a name, a description, units (when applicable), and a data type (float, integer, categorical, text, Boolean, URL). The actual data values are called assay results and are linked to the assay, the assay component, and the original data-collection–specific substance. All of the data for an assay can be represented as a table with one row per chemical and one column per assay component.

To be included in ACToR, a data source must meet several criteria: *a*) data must be publicly available; *b*) information sources must have a significant overlap with chemicals of interest; *c*) information must be indexed by chemical, that is, available on a chemical-by-chemical basis; and *d*) information must be indexed by CASRN (although data are also included for substances having no assigned CASRN). We do not require that data be peer reviewed, although for the analysis we report here, most of the data sources either have been externally peer reviewed or, when from government agencies, have undergone extensive internal review. Data entered into ACToR undergo a limited quality control process. Data are preferably taken from sources of high-quality data, so our quality control is limited to checking that the data are correctly transferred from the source via a reformatting and loading process into the ACToR database. No checks are made on the correctness of the data from the original source. Each data set is manually spot-checked for gross issues with reformatting. All CASRNs in the database are checked to be sure that they have a proper checksum (Chemical Abstracts Service 2008). (The checksum is the result of a particular formula performed on all but the final digit of the CASRN. This result must match the final digit.) All data-handling tasks are documented in standard operating procedures to ensure consistency.

The ACToR database is implemented using MySQL. Software to preprocess and load data is written in Perl, and the Web interfaces are written in Java. The use of 100% open-source software allows the entire system to be easily distributed to other interested groups. We used the ACToR database version 2008Q2d for all of the analyses in this article. Of the subsets of data sources in ACToR, only the ones most relevant to toxicology are included in this analysis and publication. ACToR is available online (http://actor.epa.gov).

## Results

### *In vivo* toxicology data

This section describes the overlap between the target chemical set and the set of toxicity data sources. Table 3 summarizes the overlap matrix. Each cell provides the number and the percentage of the 9,912 for the chemicals that have information for a specific category of data (e.g., tabular) and a particular phenotype (e.g., carcinogenicity). The last column gives the number and percentage of chemicals for each phenotype, regardless of the information category. Chemicals are only counted once in any cell, even if they have multiple data points or sources of data. Cells that list 0 indicate that there were no corresponding data from any source. The available toxicity data almost all derive from animal studies, because essentially no experimental human data are available. However, some of the data are in the form of human reference doses, or summary calls of the form “this chemical is considered to be a human carcinogen.” These data points were, of course, derived by extrapolating from primary data on animals. Chemical hazard has been evaluated for 5,810 (58.6%) of these chemicals. Carcinogenicity potential for 2,579 (26%) of these chemicals has been evaluated by at least one source. The genotoxic potential of 2,724 (27.5%) of the chemicals has been evaluated. A total of 2,862 (28.9%) of the chemicals have their developmental toxicity reported, and 1,081 (10.9%) have reproductive toxicity data reported. Food safety information (from one of the sources mentioned above) is available for 2,258 (22.8%) of the chemicals. Chemicals count in this table whether they have positive or negative data for toxicity for a particular phenotype. To date, we have not systematically tabulated the relative number of toxic and nontoxic indications for all chemicals.

Table 4 provides overlaps of the chemicals of interest with more general information and biological assays of potential interest. One or more *in vitro* biochemical assays are available for 781 (7.9%) of the chemicals. Most of these are *in vitro* cytotoxicity assays in PubChem, but also include receptor binding and enzyme inhibition data. A small number of the target chemicals (234 or 2.4%) are naturally occurring human metabolites, based on data from the Human Metabolome Database (Wishart et al. 2007).

The highest-quality toxicity assessments, based on guideline studies or on extensive review of the literature, are U.S. EPA OPP reviews (which are captured in the ToxRefDB database), U.S. EPA IRIS assessments, NTP studies, OECD SIDS guideline studies of HPV chemicals, studies in the U.S. EPA HPVIS, and assessments by the ATSDR and IARC. From the current list, there are 431 (4.3%), 536 (5.4%), 1,168 (11.8%), 343 (3.5%), 992 (10%), 216 (2.2%), and 537 (5.4%) chemicals in these respective sets (Table 4). Looking across all of these data sources, 2,767 (27.9%) are covered by one or more of these high-quality toxicology sources. Finally, a total of 4,641 (46.8%) are currently subject to one or more U.S. EPA regulations. These regulations are available online (http://www.epa.gov/ncct/toxcast/).

### Chemical categories

Both the U.S. HPV Challenge and the OECD HPV programs encourage the use of categories because of the large number of chemicals being assessed. Using a category approach, chemicals are evaluated as a group, or category, rather than as individual chemicals, and not every chemical needs to be tested for every end point. The category approach entails grouping chemicals with similar structures, physical–chemical properties, fate parameters, and toxicologic properties in order to extrapolate toxicologic information from tested chemicals and end points to untested chemicals and end points. For most categories, the number of chemicals with toxicology data that could be used for model building is much smaller than the total number of chemicals included within the category.

ACToR includes listings of chemical categories taken from the U.S. EPA HPVIS and from the OECD HPV Programme. From these lists, a total of 1,274 (12.9%) chemicals are in at least one category, and there are 256 unique categories that include at least one of the target chemicals. However, most of the categories in HPVIS represent “proposals,” which are currently under review by the U.S. EPA, such that the final number of categories and chemicals assigned to them is subject to change. In addition, the U.S. EPA is currently using chemical clustering techniques with the goal of creating chemical categories to facilitate hazard assessment of MPV chemicals. The outcome of these efforts will be included in ACToR in the future. Information will also flow in the opposite direction; that is, the data and information included in ACToR will be useful in reviewing and refining the U.S. EPA’s HPV and MPV categories.

### Production volumes

An important component of any prioritization program will be an assessment of potential for exposure. In the absence of specific information and for screening and prioritization purposes, production volumes are often used as a surrogate for exposure potential. Table 5 lists counts for each of the production volume categories. A total of 5,939 (59.9%) of the target chemicals have production volume information in the 2002 IUR.

### Properties related to chemical structure

Physical–chemical properties were calculated using the EPISuite collection of programs, which use chemical structure (in the form of a SMILES string) as input. Of the 7,099 chemicals for which structures and SMILES data were available, EPISuite was able to process 5,857. The chemicals for which calculations could not be performed were mainly certain types of salts, inorganic compounds, organometallics, or chemicals with nonstandard SMILES.

Several parameters will be useful for determining whether a compound can be bioavailable or whether it will be amenable to HTS assays: MW, logP, solubility, and vapor pressure. Typical ranges for properties for chemicals that can be tested using HTS methods are MW < 500 Da, logP between 0 and 6, and vapor pressure < 10 mm (not volatile at room temperature). Filtering the larger list against this set of criteria yields a set of 3,060 compounds that are candidates for HTS testing. One could produce slightly different lists, of course, by altering these threshold values. The primary requirements for use in an HTS assay are that chemicals be soluble in dimethyl sulfoxide or water, that they be nonvolatile, and that they be stable in solution.

**Figure 1:**
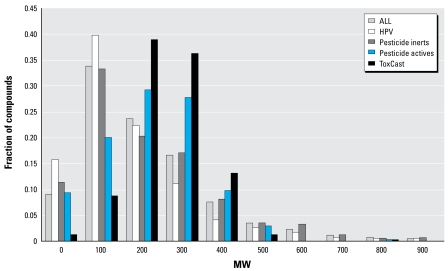
Distribution of MW for representative chemical sets. The sum of fractions for each data set equals 1.

**Figure 2:**
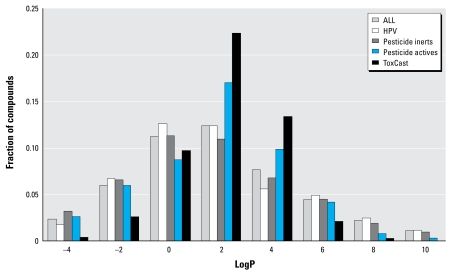
Distribution of calculated logP for representative chemical sets. The sum of fractions for each data set equals 1.

Figures 1 and 2 show distributions of MW and logP for the complete set of chemicals with structures and for four representative subsets of the larger data collection: HPV chemicals, pesticide inert ingredients, pesticide active ingredients, and the ToxCast phase I collection. For MW, the main trend is that the HPV and pesticide inert collections contain significantly larger fractions of low-MW chemicals (< 200 Da) than do the pesticide active ingredients and the ToxCast chemicals. Given that most ToxCast phase I chemicals are pesticide active ingredients and that this set was prefiltered for HTS suitability, it is not surprising that this set has a smaller fraction of high-MW chemicals (> 500 Da) than do the other collections. Distributions of logP are similar for all of the subsets except for ToxCast, which is more tightly clustered, with a peak between 0 and 2.

## Discussion

In this article we describe and analyze a compilation of chemical structures, physical–chemical properties, *in vitro* biochemical assay data, and *in vivo* toxicology data on a large collection of chemicals of interest to the U.S. EPA. Most of these data are currently publicly available but have not been organized previously in a unified manner that allows for the analysis of large trends and simplified review based on either chemical or assay axes. The data we describe here are a subset of those contained in the ACToR system being developed at the U.S. EPA to manage large collections of data on environmental chemicals.

We have used the ACToR database to characterize the state of toxicologic knowledge on a subset of environmental chemicals that are on a variety of lists of interest to the U.S. EPA. This analysis is used to address the extent of the perceived data gap on potentially toxic chemicals. Although the picture is complicated, some summary observations are possible. About two-thirds of the chemicals have some toxicology information. The unique set of chemicals in Table 3 is 6,551 of 9,912 (66%). The alternative view is that many of these chemicals remain largely uncharacterized—a total of 3,361 (34%) chemicals have no information in any of the data sources we used in this analysis. On the other hand, more than one-quarter (27.9%) have been analyzed in one or more high-quality and/or systematic evaluation programs (NTP, IRIS, ToxRefDB, U.S. EPA HPV, OECD SIDS, IARC, and/or ATSDR). Of the individual types of toxicity (or end points) that have been tabulated, carcinogenicity, genotoxicity, and developmental and reproductive toxicity have been most widely covered (26%, 27.5%, 28.9%, and 10.9%, respectively).

One immediate application of this analysis is to select compounds for further screening in programs such as ToxCast. ToxCast phase I is using a set of compounds (primarily pesticide active ingredients) that are amenable to HTS and that have rich toxicologic data. The outcome of the phase I analyses will be a set of “signatures” that use *in vitro* screening data as inputs to predict *in vivo* toxicology phenotypes with high enough sensitivity and specificity to be useful for prioritization for more detailed testing. Phase II needs to include compounds that can be used to independently validate the phase I signatures. Therefore, the phase II set of chemicals should contain as many compounds as possible with high-quality *in vivo* toxicology data, have physical–chemical properties that make them candidates for HTS, and be drawn from a more diverse collection than the phase I chemicals to help define the chemical domain of applicability of the signatures. We calculated the intersection of the set of 2,767 chemicals that have data from one of the high-quality and/or systematic toxicology data sources (NTP, IRIS, HPVIS, OPP/ToxRefDB, OECD SIDS, IARC, ATSDR) with the set of 3,060 chemicals with reasonable physicochemical properties. This yields a list of 1,308 candidate chemicals that have both high-quality toxicity data and physicochemical properties very well suited for HTS. After removing the ToxCast phase I chemicals, we arrived at a list of 1,046 chemicals that are candidates for inclusion in ToxCast phase II for use in validating ToxCast phase I findings across a variety of end points. Many of these chemicals are currently being analyzed in a series of HTS assays at the NIH Chemical Genomic Center (NCGC) as part of the Tox21 partnership between U.S. EPA, NCGC, and NTP. These Tox21 chemicals include an even broader range of physicochemical properties, with a MW range of 32 to 1,255 and a logP range of −13.2 to 13.2. An important analysis that is yet to be carried out is chemical structure characterization and clustering for the ToxCast phase I and II lists and the larger target list. This will be important to help understand our ability to extrapolate within and across chemical structural classes.

ACToR is not alone in its goal of aggregating large sets of chemical structure and assay data but is distinguished from other efforts by its focus on toxicology and environmental chemicals and its goal of facilitating computational analysis. PubChem (NCBI 2008) is the largest effort currently available, with information on more than 10 million unique chemical compounds. ChemSpider (2008) is an even larger chemical aggregation project but does not house biological data or downloadable data sets. Another important comparison is with TOXNET, which is a collection of multiple data sources covering many aspects of chemical toxicity. TOXNET has a common search engine that allows the user to easily find data from multiple sources. However, it is a closed system that does not allow a user to pull together data sets that are useful for computational purposes. One unique aspect of the ACToR system is that it aggregates the data from PubChem (focused on chemical structure and HTS *in vitro* assay data) and TOXNET (NLM 2008) (focused on *in vivo* toxicology data) and combines it in a way that it can be used for computational analysis. eChemPortal (OECD 2008) is an OECD effort very similar to ACToR. It mainly aggregates information on HPV chemicals and pesticides. eChemPortal currently contains links to seven large database systems, some of which contain what in ACToR are multiple individual databases (e.g., INCHEM contains 11 individual databases; International Programme on Chemical Safety 2008). Unlike eChemPortal, which provides links to Web pages for the component databases, ACToR extracts tabular data from a large number of sources and makes it searchable by name, CASRN, or chemical structure. A system called Vitic is being developed by Lhasa Limited in collaboration between the European Chemicals Agency’s International Uniform Chemical Information Database (IUCLID 2008) project and a number of pharmaceutical companies, with the goal of being an international toxicology information center (Judson et al. 2005). In addition, the European Substances Information System provides links to a number of databases, including U.S. EPA HPV, IUCLID, and European Inventory of Existing Commercial Chemical Substances. Finally, the Chemical Effects in Biological Systems project at the National Institute of Environmental Health Sciences is constructing a multidomain information repository to hold the detailed results and summaries of *in vivo* and *in vitro* toxicology experiments from NTP studies, with particular emphasis on toxicogenomics and microarray experiments (Waters et al. 2008).

To adequately characterize the toxicology of all environmental chemicals of potential concern, we still face significant challenges. Screening and prioritization approaches such as ToxCast can make significant headway in analyzing small organic and organometallic compounds, for which most HTS methods have been developed for use in the pharmaceutical industry. Because of solubility and volatility issues, however, many exceptionally high-and low-MW environmental compounds or highly lipophilic compounds may require new screening methods. Of special interest are nano-materials, which will require new standards for description (i.e., size, shape, composition, etc.) and may require entirely new approaches to thinking about cellular and organism-level toxicity (Maynard et al. 2006; Shaw et al. 2008). One rarely has knowledge of metabolites that can arise from a parent compound *in vivo* and whether any of these metabolites are more or less toxic than the parent. However, a number of metabolic pathway databases and/or simulators are currently available or under development that could potentially be incorporated into ACToR in the future. Finally, a large number of known biological pathways (i.e., signaling, metabolism, etc.) have the potential to lead to toxicity when significantly perturbed. Many toxicity pathways have been implicated in whole-animal end points, such as liver cancer, and most chemicals can perturb multiple candidate toxicity pathways. Gaining a predictive and mechanistic understanding of chemical toxicity will require the ability to predict which set of toxicity pathways are triggered by individual chemicals.

A significant amount of data on chemicals is not currently accessible for modeling, either because it is not publicly available or because it is not yet extracted from primary reports in a useful, tabular format. Several efforts are under way at the U.S. EPA and other institutions to extract, standardize, compile, and analyze such high-quality data (U.S. EPA 2008b). We would welcome collaborations with other groups producing such tabular data sets on these important classes of chemicals.

## Conclusions

In this article, we have described a process for determining a set of environmental chemicals with the highest need for hazard and risk evaluation, which is based primarily on objective, simple measures of data availability. In addition, we have collected information from a large number of publicly available sources to determine the state of our current knowledge of these chemicals. The list we developed includes HPV and MPV chemicals, pesticide and antimicrobial active and inert ingredients, and potential air and drinking water pollutants, in addition to chemicals already being evaluated by the U.S. EPA IRIS and ToxCast programs. Although the input lists are developed from the perspective of regulatory and research needs of the U.S. EPA, we believe that our overall conclusions will have wide applicability. This process resulted in a collection of 9,912 unique chemicals. We have at least limited hazard information on approximately two-thirds of these and detailed toxicology information on approximately one-quarter. The combination of chemical structure and *in vivo* data on this large range of environmental chemicals in ACToR can facilitate structure–activity relationship and other types of trend analyses. These analyses will have direct relevance to U.S. EPA programs such as HPV Challenge (U.S. EPA 2008m) and the Chemical Assessment and Management Program (U.S. EPA 2008b).

The principal reason for the lack of more complete toxicity information is the extremely high cost for full evaluation using standard guideline animal studies, which is millions of dollars per chemical. This has prompted the call for the use of more cost-effective HTS methods for quickly screening and prioritizing chemicals for more detailed testing. The analysis presented here is a first step in such a screening and prioritization process being carried out at the U.S. EPA as part of the ToxCast program. ToxCast is using hundreds of *in vitro* HTS assays to assess potential mechanisms through which chemicals could cause toxicity. This hazard prediction is just one of several axes along which potential risk needs to be evaluated. Chemicals need to be evaluated for exposure potential, and for adsorption, distribution, metabolism, and excretion (ADME) and pharmacokinetics properties. Of special concern would be compounds that are persistent or bioaccumulative. Researchers at Health Canada have demonstrated a process to evaluate exposure for many of these chemicals (Health Canada 2006). Chemical structure analysis can be used as part of the prioritization process, for instance, in predicting bioaccumulation potential (Meylan et al. 1999; Weisbrod et al. 2007) and fractional absorption (Ekins et al. 2007a, 2007b). Nonanimal experimental methods are available to approximate gut absorption (Sun et al. 2008) and total hepatic clearance (Naritomi et al. 2003). Reverse-pharmacokinetic methods (Brightman et al. 2006) can be used to predict oral doses that would be required to trigger molecular processes, for instance, based on half maximal inhibitory concentrations (IC_50_) for receptor binding from *in vitro* assays. These and other related approaches are being considered as part of the overall ToxCast screening and prioritization process. Of special relevance to the ToxCast program, we have identified a set of 1,046 candidate chemicals that have reliable *in vivo* toxicology data and have physico-chemical properties that make them suitable for *in vitro* HTS analysis. These are candidates for phase II of the ToxCast program, which will be used to validate *in vitro*-to-*in vivo* toxicity predictions, which are one outcome of phase I of this program.

Another important input to this process is high-quality, tabular *in vivo* toxicity data. This is required to anchor our *in vitro*-to-*in vivo* prediction models, in both the model building and model validation phases. Initially, we are making use of the results of guideline toxicology studies for pesticide active ingredients, which are being collected into the U.S. EPA ToxRefDB (Martin et al. 2008). We are expanding this data collation effort in coordination with the ACToR project. As already described, ACToR is a database consisting of information on environmental chemicals from a wide number of sources. However, currently much of the high-quality toxicology data indexed in ACToR still resides in text reports and remains to be manually extracted into tabular form.

An important aspect of this program is openness and transparency. The ToxCast program is making all of its data publicly available. It has a large community of collaborators, from government labs, companies, and universities. Finally, important open venues for learning about this program and the Chemical Prioritization and Exposure Communities of Practice are providing input (U.S. EPA 2008i). These are bringing together representatives from U.S. EPA, state, and other national environmental regulatory organizations, academic labs, stakeholder companies, and public interest groups, all of whom are providing important input as we collectively work to address this important problem. All of these efforts are consistent with achieving the goals and vision of the recent NRC report *Toxicity Testing in the 21st Century* (NRC 2007).

## Figures and Tables

**Table 1:** Numbers of chemicals that overlap between the screening target chemical collections.

		EPA CCL1	EPA CCL2	EPA draft CCL3	EPA PCCL	EPA DWSHA	EPA EDSP 73	EPA GLNPO PBT	EPA HAPs	EPA HPV	EPA HPV Challenge	EPA HPVIS	EPA IRIS	EPA IRIS nominations	EPA IRIS queue	EPA IUR (2002)	EPA OPPIN pesticide active	EPA OPPIN antimicrobial active	EPA OPPIN food use	EPA OPPIN inerts	EPA inerts nonfood	EPA TRI	ToxCast phase I	ToxRefDB
		47	39	92	528	200	73	429	185	2,539	1,973	992	535	20	68	5,375	834	337	1,320	3,532	3,492	636	308	431
EPA CCL1	47	47	39	13	34	25	6	0	14	11	11	4	31	2	7	14	15	5	14	8	6	27	11	16
EPA CCL2	39	39	39	13	28	19	5	0	12	10	10	4	25	1	5	13	13	4	13	7	5	22	10	15
EPA draft CCL3	92	13	13	92	92	19	9	2	28	29	30	8	56	1	10	39	31	11	41	19	10	60	25	31
EPA PCCL	528	34	28	92	528	62	33	21	77	237	259	91	187	6	27	302	125	52	166	162	135	206	73	93
EPA DWSHA	200	25	19	19	62	200	29	4	69	61	60	26	176	6	40	77	63	23	69	55	33	130	43	59
EPA EDSP 73	73	6	5	9	33	29	73	1	12	8	11	6	57	1	3	12	64	15	66	15	12	44	56	66
EPA GLNPO PBT	429	0	0	2	21	4	1	429	8	109	75	37	22	2	4	194	3	2	12	43	39	20	4	7
EPA HAPs	185	14	12	28	77	69	12	8	185	92	101	27	144	3	36	122	24	15	43	68	43	173	15	21
EPA HPV	2,539	11	10	29	237	61	8	109	92	2,539	1,746	701	145	11	34	2,187	102	84	246	720	676	162	13	28
EPA HPV Challenge	1,973	11	10	30	259	60	11	75	101	1,746	1,973	703	147	10	37	1,759	77	60	212	612	567	166	11	25
EPA HPVIS	992	4	4	8	91	26	6	37	27	701	703	992	54	6	12	747	37	34	81	268	250	58	8	15
EPA IRIS	535	31	25	56	187	176	57	22	144	145	147	54	535	10	50	183	179	42	187	115	75	290	122	147
EPA IRIS nominations	20	2	1	1	6	6	1	2	3	11	10	6	10	20	0	13	2	2	4	6	5	9	1	2
EPA IRIS queue	68	7	5	10	27	40	3	4	36	34	37	12	50	0	68	46	8	8	10	31	18	46	2	4
EPA IUR (2002)	5,375	14	13	39	302	77	12	194	122	2,187	1,759	747	183	13	46	5,375	151	140	378	1,195	1,126	230	23	47
EPA OPPIN pesticide active	834	15	13	31	125	63	64	3	24	102	77	37	179	2	8	151	834	217	484	178	169	175	272	363
EPA OPPIN antimicrobial active	337	5	4	11	52	23	15	2	15	84	60	34	42	2	8	140	217	337	129	155	151	57	33	63
EPA OPPIN food use	1,320	14	13	41	166	69	66	12	43	246	212	81	187	4	10	378	484	129	1,320	744	724	169	239	300
EPA OPPIN inerts	3,532	8	7	19	162	55	15	43	68	720	612	268	115	6	31	1,195	178	155	744	3,532	3,183	136	22	35
EPA inerts nonfood	3,492	6	5	10	135	33	12	39	43	676	567	250	75	5	18	1,126	169	151	724	3,183	3,492	92	15	26
EPA TRI	636	27	22	60	206	130	44	20	173	162	166	58	290	9	46	230	175	57	169	136	92	636	112	144
ToxCast phase I	308	11	10	25	73	43	56	4	15	13	11	8	122	1	2	23	272	33	239	22	15	112	308	304
ToxRefDB	431	16	15	31	93	59	66	7	21	28	25	15	147	2	4	47	363	63	300	35	26	144	304	431

**Table 2:** Categories of assays in ACToR that are described in this analysis.

Assay category	Description	Examples
Physical–chemical	Physical and chemical properties (*in vitro* and/or *in silico*)	MW
		LogP
		Boiling point
Biochemical	Biochemical (non-cell-based) (*in vitro* and/or *in silico*)	Enzyme inhibition constants
		Receptor binding constants
*In vivo* toxicology (tabular)	Tabulated results from primary or secondary animal-based studies of chemical effect	Clinical chemistry
		Histopathology
*In vivo* toxicology (study listing primary)	Primary studies are available but have not been tabulated	Clinical chemistry
		Histopathology
		Developmental and reproductive assays
*In vivo* toxicology (summary calls)	Derived summary determinations of risk	Chemicals determined to pose a defined risk of human cancer
*In vivo* toxicology (summary report via URL)	Links to text reports on the Web for which specific data values are not directly accessible in tabular form	Reports from U.S. EPA IRIS or NTP
Regulatory	Listings of chemicals that fall under specific environmental laws or government mandates	TSCA

**Table 3:** Summary of overlap between the target chemical list and the set of assay components.

Assay	Tabular	Primary study listing	Summary calls	Summary report via URL	Any
Hazard	4,454 (44.9)	0	255 (2.6)	4,767 (48.1)	5,810 (58.6)
Carcinogenicity	1,211 (12.2)	401 (4.0)	726 (7.3)	2,035 (23.3)	2,579 (26)
Genotoxicity	2,496 (25.2)	1,102 (11.1)	32 (0.3)	1,047 (10.6)	2,724 (27.5)
Developmental toxicity	755 (7.6)	37 (0.4)	125 (1.3)	2,324 (23.4)	2,862 (28.9)
Reproductive toxicity	734 (7.4)	0	31 (0.3)	396 (4)	1,081 (10.9)
Food safety	1,692 (17.1)	0	533 (5.4)	0	2,258 (22.8)

Each cell provides the number and the percentage of the 9,912 for the chemicals that have information for a specific category of data (e.g., tabular) and a particular phenotype (e.g., carcinogenicity). The last column gives the number and percentage of chemicals for each phenotype, regardless of the information category. Chemicals are only counted once in any cell, even if they have multiple data points or sources of data. Cells with 0 indicate that there were no corresponding data from any source.

**Table 4:** Coverage by specific data types and sources.

Name	Total	Percent coverage
Biochemical	781	7.9
Human-metabolite	234	2.4
ToxRefDB	431	4.3
IRIS	536	5.4
NTP	1,168	11.8
SIDS	343	3.5
HPVIS	992	10.0
ATSDR	216	2.2
IARC	537	5.4
ToxRefDB, IRIS, NTP, SIDS, ATSDR, and/or IARC	2,767	27.9
Regulation	4,641	46.8

**Table 5:** Production volumes from the 2002 IUR.

Production volume (lb/year)	Count	Percent coverage
< 10K	11	0.1
10K–500K	2,827	29.0
> 500K–1M	485	4.9
> 1M–10M	1,381	14.0
> 10M–50M	512	5.0
> 50M–100M	130	1.0
> 100M–500M	246	2.0
> 500M–1B	67	0.7
> 1B	280	3.0
Total	5,939	60.0

Abbreviations: B, billion; K, thousand; M, million.
